# The effect of different cleansing methods for removing temporary cement on the tensile bond force of permanently cemented implant‐supported metal copings: An in vitro study

**DOI:** 10.1002/cre2.593

**Published:** 2022-05-26

**Authors:** Behnaz Ebadian, Mohammad Jowkar, Amin Davoudi, Amirhossein Fathi, Mohsen Ziaei, Einar Berg

**Affiliations:** ^1^ Department of Prosthodontics, Dental Implants Research Center Dental Research Institute, School of Dentistry, Isfahan University of Medical Sciences Isfahan Iran; ^2^ Department of Prosthodontics Isfahan University of Medical Sciences Isfahan Iran; ^3^ Department of Prosthodontics Shahrekord University of Medical Sciences Shahrekord Iran; ^4^ Department of Prosthodontics Dental Materials Research Center, Isfahan University of Medical Sciences Isfahan Iran; ^5^ Department of Prosthodontics Bergen University Bergen Norway

**Keywords:** CAD/CAM, cleansing method, recementation, tensile bond force

## Abstract

**Objectives:**

Complete cleaning of temporary cement before permanent cementation of cement‐retained implant‐supported prosthesis (CISP) when recementing the crown is critical. This study evaluated the effect of different cleaning methods for removing traces of temporary cement on the final tensile bond force (TBF) of CISP recemented with resin cement.

**Materials and Methods:**

Seventy computer‐aided design/computer‐aided manufacturing metal implant‐supported copings were prepared and distributed into seven groups (*N* = 10). Copings of six groups (60 samples) were cemented with temporary cement with eugenol and subjected to 5000 thermocycling. After debonding by a universal testing machine, the internal surfaces of the copings were cleaned using one of the six following methods: 1‐an ultrasonic water bath (UW), 2‐sandblasting, then washing with water (SW), 3‐sandblasting and an ultrasonic water bath (SUW), 4‐an ultrasonic isopropyl alcohol bath (UA), 5‐sandblasting, then washing with isopropyl alcohol (SA) or 6‐sandblasting and an ultrasonic isopropyl alcohol bath (SUA). Then the subjects were subsequently cemented by dual‐cure self‐adhesive resin cement. In the seventh group (control, *N* = 10), the copings were cemented by dual‐cure self‐adhesive resin cement without the temporization phase. The TBF was tested using a universal testing machine with a cross‐head speed of 1 mm/min. Two‐way analysis of variance (ANOVA) and post‐hoc Tamhane tests were used for statistical analysis at a significance level of α = .05.

**Results:**

The maximum mean of TBF value was observed in SUA group (845 ± 203 N), and the minimum was observed in the temporary cement group (49 ± 20 N). All groups which were cleaned with isopropyl alcohol showed significantly higher TBF values compared with those cleaned with water.

**Conclusions:**

Cleaning of the inner surface of metal copings after debonding with sandblasting and isopropyl alcohol results in the highest value of TBF by eliminating the effect of remaining eugenol and removing traces of temporary cements.

## WHAT IS KNOWN

Recent studies have a controversy on the best cleansing method of remnant temporary cement of cemented implanted‐supported crowns before final cementation.

## WHAT THIS STUDY ADDS

This study suggests that cleaning with sandblasting and isopropyl alcohol may be a promising method for the cleansing of removing traces of temporary cement. This cleansing method provides enhanced final tensile bond force of implant‐supported metal copings.

## 1 INTRODUCTION

Implant‐supported fixed dental prostheses can be screw‐retained or cement‐retained. When screw‐retained, a passive fit of the prosthesis is crucial as there is no cement layer to compensate for misfit‐induced strain (Romanos et al., 2019). A cement‐retained implant‐supported prosthesis (CISP) has the advantage of passive fit and occlusal integrity (Davoudi & Rismanchian, 2019; Hamed et al., 2020; Romanos et al., 2019). Therefore, cementation of the implant‐supported prosthesis is a critical procedure (Hamed et al., 2020; Romanos et al., 2019). It has been demonstrated that different factors can influence the efficacy of the bond strength of such cemented prostheses (Malpartida‐Carrillo et al., 2020). Abutment material, height and convergence angle, adaptation and marginal accuracy, surface pretreatment, cement type, and cement degradation rate are some of these factors (Jain et al., 2018; Malpartida‐Carrillo et al., 2020). Temporary cements, which are the most commonly used luting agents used for CISP (Almehmadi et al., 2019), should be strong enough to attain retention of the prosthesis, yet weak enough to allow the clinician to retrieve the CISP when needed (Almehmadi et al., 2019). Nevertheless, in some situations, like insufficient abutment height, it may be necessary to choose a luting agent with higher bonding strength (Gómez‐Polo et al., 2018). Also, in cases of removed temporarily cemented implant‐supported restorations, clinicians may prefer to use a permanent cement in the recementation procedure to increase retention force (Almehmadi et al., 2019).

Studies on the tensile bond force (TBF) of different luting agents are available (Almehmadi et al., 2019; Garg et al., 2014; Sheets et al., 2008). Resin‐based luting agents are have been proposed because they provide high retentive values with low microleakage (Almehmadi et al., 2019). In such cases, it is critical to remove previous temporary cement from the intaglio surface of the CISP to eliminate any possible negative interaction of remaining eugenol with resin‐based luting cement (Almehmadi et al., 2019; Woody & Davis, 1992). Different methods have been tested to determine the best one for cleaning and removing temporary cement residues from the inner surface of the crowns (Song et al., 2019; Woody & Davis, 1992). Among these are mechanical removal with an excavator or scalpel, sandblasting with air‐borne abrasive particles, ultrasonic cleaning, and the use of chemical solvents. However, most of these methods have been tested on tooth‐supported restorations (Ayad et al., 1998; Song et al., 2019; Woody & Davis, 1992; Zhang et al., 2004).

The efficacy of different cleaning methods for removing temporary cement residues from the CISP and their role in the final TBF of CISP has not yet been investigated. The aim of this study was to observe the effect of six different mechanical cleaning methods in combination with water or isopropyl alcohol on the retentive force of CISP. The defined null hypothesis was that neither cleaning methods nor solutions affect the TBF of CISP.

## 2 MATERIALS AND METHODS

### 2.1 Preparation of analogs and abutments

**Figure 1:**
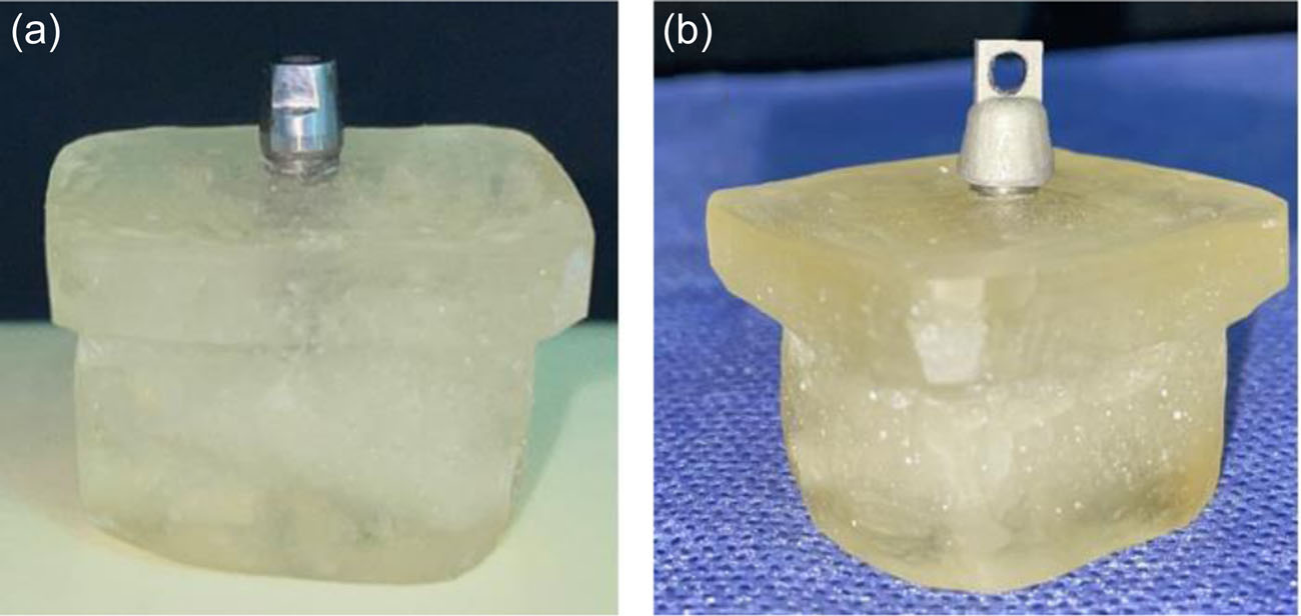
(a) Prepared abutment screwed on analog mounted in a self‐cure acrylic resin block, (b) Co‐Cr coping with the occlusal loop on the prepared abutment

Seventy implant analogs (OPR, Zimmer, SwissPlus, Carlsbad, CA, USA) were vertically mounted, each in its own self‐cure acrylic resin block (6 × 10 × 20 mm). The analog's alignment was verified by a surveyor. The blocking surface was placed 1 mm below the abutment‐analog junction. Seventy abutments (FMS, Zimmer, SwissPlus, θ 4.8, Carlsbad, CA, USA), shortened to 5 mm in height, were screwed onto the analogs with a 30 Ncm torque force by means of a calibrated prosthetic torque wrench (Zimmer Dental, Carlsbad, CA, USA) (Figure 1) and subjected to the testing procedures as described below.

### 2.2 Preparation of copings

**Figure 2:**
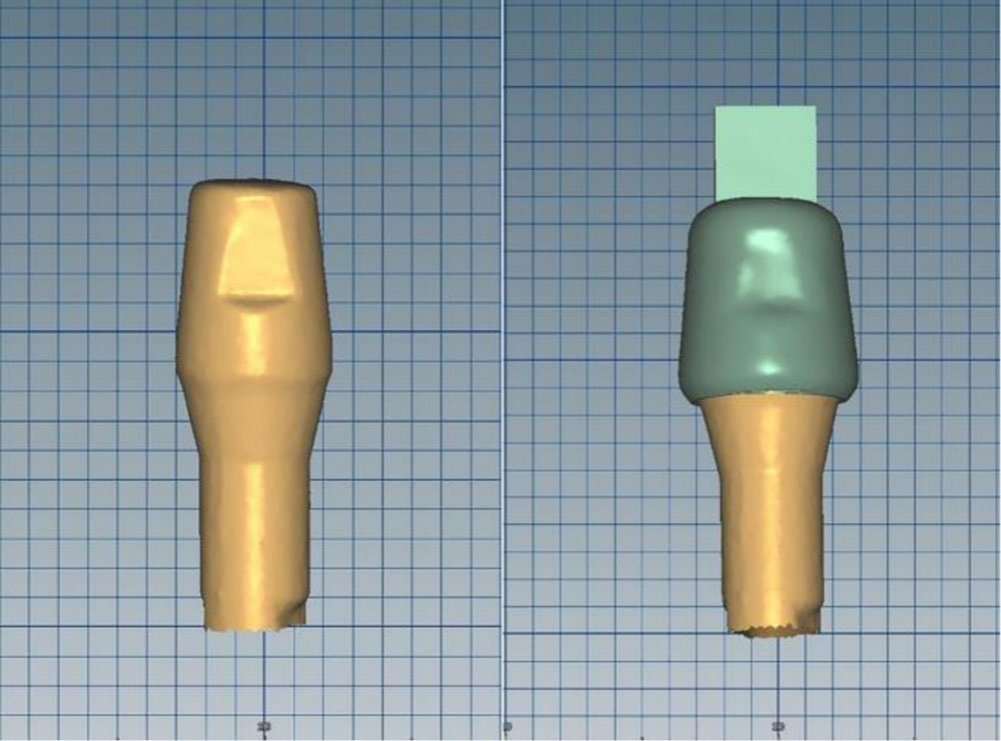
Designed coping with an occlusal projection on the scanned abutment

Each abutment was scanned individually (Figure 1) and 70 Co‐Cr copings (Ceramill Sintron, Amann Girrbach, North America, Charlotte, USA) were fabricated using a computer‐aided design/computer‐aided manufacturing (CAD/CAM) device (Amann Girrbach, North America, Charlotte, USA) with a 30 µm space for the luting cement. Each coping was prepared with an occlusal loop which was drilled after milling to provide a suitable grip for a tensile test in the universal testing machine (Figure 2).

The marginal fit was evaluated at ×4 magnification under a stereomicroscope, and copings with improper fit were excluded.

All abutments and copings were cleaned in an ultrasonic bath containing 96% ethanol, for 5 min at 30°C, subsequently washed with distilled water, and then dried. The screw access was filled with Teflon.

### 2.3 Experimental conditions

The 70 copings were divided into six experimental groups and one control group (*n* = 10). The experimental groups were prepared and subjected to different cleaning treatments of the internal surfaces of the copings; the seventh group (C) served as a control. The groups are defined in Table 1.

**Table 1:** Definition of groups

Groups	N	Cleaning method
C	10	No special cleaning. Copings cemented with Panavia SA luting plus (Kuraray, Kurashiki, Japan)
UW	10	Copings immersed in ultrasonic water bath at 30° C for 15 min, washed with distilled water for 30 s, dried
SW	10	Copings sandblasted with 50 µm aluminium oxide particles (1.5 bar, 15 s, applied at a distance of 1 cm and 45° to the nozzle), washed with distilled water for the 30 s, dried
SUW	10	Copings sandblasted with 50 µm aluminium oxide particles (1.5 bar, 15 s, applied at a distance of 1 cm and 45° to the nozzle), washed with distilled water for 30 s, immersed in ultrasonic water bath at 30° C for 15 min, washed with distilled water for 30 s, dried
UA	10	Copings immersed in ultrasonic isopropyl alcohol bath at 30° C for 15 min, washed with distilled water for 30 s, dried
SA	10	Coping sandblasted with 50 µm aluminium oxide particles (1.5 bar, 15 s, applied at a distance of 1 cm and 45° to the nozzle), washed with isopropyl alcohol for 30 s, washed with distilled water, dried
SUA	10	Copings sandblasted with 50 µm aluminium oxide particles (1.5 bar, 15 s, applied at a distance of 1 cm and 45° to the nozzle), washed with distilled water for 30 s, immersed in ultrasonic isopropyl alcohol bath at 30° C for 15 min, washed with distilled water for 30 s, dried

Abbreviations: C, control; SA, sandblast and washed with isopropyl alcohol; SW, sandblast and washed with water; SUA, sandblast and ultrasonic with isopropyl alcohol; SUW, sandblast and ultrasonic with water; Temp, temporary cement group; UA, ultrasonic with isopropyl alcohol; UW, ultrasonic with water.

The copings of Group C were cemented with Panavia SA luting plus (Kuraray, Kurashiki, Japan) according to the manufacturer's instructions. Temporary cement (TempBond, Kerr, Hamm, Germany) was used for cementation of the other six groups (60 samples), using the following procedure: All internal walls of the copings were covered with cement with a brush and pressed down for 10 s by hand. Then the specimens were loaded by a 5 kg force for 10 min according to American Dental Association specification No. 96. Excess cement was removed with a dental explorer before the complete setting.

When the cementation procedure was completed, all samples were immersed in 37°C distilled water for 24 h. To simulate the intraoral environment, the samples were undergoing 5000 thermal cycles at 5–55°C with 30 s of dwell time.

The copings of all groups were pulled out at a cross‐head speed of 1 mm/min in a universal testing machine (Type LFM‐L, Walter+Bai AG, Löhningen, Switzerland) and the TBF values were recorded.

After decementation of the experimental groups, the internal surfaces of all abutments were mechanically cleaned with a dental excavator so that no cement particles were seen with a naked eye and subsequently cleaned using dental polishing prophy brushes (Kerr, Hamm, Germany) and CleanPolish paste (Kerr, Hamm, Germany) for 1 min. Thereafter, the copings of the experimental groups were prepared as described in Table 1.

All copings in the experimental groups were subsequently cemented with Panavia SA luting plus (Kuraray, Kurashiki, Japan) resin cement according to the manufacturer's instructions. When the cementation procedure was completed, all samples were immersed in 37°C distilled water for 24 h. To simulate the intraoral environment, the samples were undergoing 5000 thermal cycles at 5–55°C with 30 s dwell time.

The copings were then pulled out in the same universal testing machine and under the same conditions as described above, and the TBF values were recorded.

### 2.4 Sample size calculations and statistical methods

The sample size was determined considering an effect size of 40 N for any measurement parameter. To achieve this effect size and 80% power, the estimated sample size per group was 10.

Levene, two‐way analysis of variance (ANOVA), and Tamhane post hoc tests were used for statistical analyses. A significance level of α = .05 was used in the calculations. All statistical analyses were performed by using a statistical software program (IBM SPSS Statistics, v24; IBM Corp, Armonk, NY, USA).

## 3 RESULTS

The highest mean TBF value was found in the SUA group (845 ± 203 N), the lowest when temporary cement was used (49 ± 20 N). Detailed TBF values of all studied groups are presented in Table 2.

**Table 2:** Mean ± SD of tensile bond force (N) of the groups

	Mean	Std. Deviation	95% Confidence interval for mean		Minimum	Maximum
			Lower bound	Upper bound		
Temp	49.6	20.5	35.8	63.4	29	88.4
C	631.7	165.7	520.4	743.1	379.5	872
UW	432.2	97.7	362.3	502.2	306.2	658.3
SW	445.3	79.3	388.6	502	351.2	574.7
SUW	458.9	91.5	393.4	524.4	247.6	558
UA	708.2	173.1	584.3	832	526.2	1043.3
SA	700.9	121.9	607.2	794.6	539.4	937.8
SUA	845.7	203.3	675.7	1015.7	534.9	1098.3

Abbreviations: C, control; SA, sandblast and washed with isopropyl alcohol; SW, sandblast and washed with water; SUA, sandblast and ultrasonic with isopropyl alcohol; SUW, sandblast and ultrasonic with water; Temp, temporary cement group; UA, ultrasonic with isopropyl alcohol; UW, ultrasonic with water.

The Levene test used to analyze the homogeneity of the collected data showed significant differences between the group variances (*p* = .00). Hence, it was appropriate to use a two‐way ANOVA test, which showed a significant difference (*p* < .001) between the two solutions used (Table 3).

**Table 3:** Two‐way ANOVA results of different methods and materials on tensile bond force

Intervention	Type III Sum of Squares	*df*	Mean square	*F*	Sig.
Methods	796075.27	2	398037.64	2.26	0.11
Solution	13257713.66	1	13257713.66	75.43	0.00
Methods × Solution	455060.45	2	227530.23	1.29	0.28

Abbreviation: ANOVA, analysis of variance.

Tamhane post hoc test was used for pair‐wise comparison of the study groups (Table 4). There were no significant differences in TBF values between the groups where isopropyl alcohol was used (ultrasonic with isopropyl alcohol [UA], sandblast and washed with isopropyl alcohol [SA], and sandblast and ultrasonic with isopropyl alcohol [SUA]). Similarly, there were no significant differences in TBF values between the groups where water was used (ultrasonic with water [UW], sandblast and washed with water [SW], and sandblast and ultrasonic with water [SUW]). All isopropyl alcohol groups showed significantly higher TBF values than all the water groups. The control group showed a significantly higher TBF value than the water groups, no different from the UA and SA groups, but a significantly lower TBF value than the SUA group.

**Table 4:** Pair‐wise comparison between study groups (Tamhane post hoc)

Groups	C	UW	SW	SUW	UA	SA	SUA
Temp	0.00	0.00	0.00	0.00	0.00	0.00	0.00
C	‐	0.01	0.03	0.06	0.87	0.93	0.01
UW	‐	‐	1.00	1.00	0.00	0.00	0.00
SW	‐	‐	‐	1.00	0.00	0.00	0.00
SUW	‐	‐	‐	‐	0.00	0.00	0.00
UA	‐	‐	‐	‐	‐	1.00	0.33
SA	‐	‐	‐	‐	‐	‐	0.30

Abbreviations: C, control; SA, sandblast and washed with isopropyl alcohol; SW, sandblast and washed with water; SUA, sandblast and ultrasonic with isopropyl alcohol; SUW, sandblast and ultrasonic with water; Temp, temporary cement group; UA, ultrasonic with isopropyl alcohol; UW, ultrasonic with water.

## 4 DISCUSSION

This study presents the effect of different cleaning methods on TBF for removing traces of temporary cement on implant‐supported CAD/CAM metal copings cemented with permanent luting cement. The findings indicate that the null hypothesis of no difference between the different methods was rejected.

TBF values of CISP reported in several studies of temporary, semi‐permanent, and permanent luting cement range from 117 N (Sheets et al., 2008) to 810 N (Garg et al., 2014), are in agreement with present TBF values.

Studies on the effect of cleaning traces of temporary cement from dental implant abutments and the inner surface of the CISP before permanent recementation is sparse. Keum et al. evaluated the effect of cleaning protocols for the removal of temporary cement on tensile bond strength (TBS) of copings cemented to implant abutments (Keum & Shin, 2013). In this study, temporary cement was removed using plastic curettes, pumice and rubber cups, and sandblasting techniques. The results indicated that plastic curettes were not effective in improving TBS of the permanently cemented prosthesis. However, the use of rubber cups with pumice or sandblasting enhanced the TBS.

In another study, Mi‐Young Song et al. examined the effect on TBS of cleaning methods for removal of traces of temporary cement on molar tooth crowns cemented with zinc phosphate and resin‐modified glass ionomer (Song et al., 2019). The cleaning techniques included orange solvent, ultrasonic baths, and sandblasting. The results indicated that while sandblasting significantly increased the TBS of crowns cemented by zinc phosphate, this procedure did not have a similar effect when resin‐modified glass ionomer cement (RMGI) were used. It was hypothesized that this was caused by the low bond strength of RMGI to metal specimens. It was also found that there was no significant difference between the ultrasonic group and the orange solvent group.

In this study, the mean highest TBF value was observed in the SUA group (845 ± 203 N), followed by groups UA, and SA, which is in agreement with the results of the above‐mentioned studies (Keum & Shin, 2013; Song et al., 2019). According to some research, sandblasting with air‐borne abrasive particles, which alters the surface by causing irregularities, enhances the interlocking of the cement and final TBS (Gurbuz et al., 2008; Rismanchian et al., 2015).

Although the present findings and previous ones (Keum & Shin, 2013; Song et al., 2019) are roughly coinciding, some noticeable differences in methodology exist. Thus, all our copings were designed and prepared by CAD/CAM technology to eliminate any potential bias or inaccuracy. Equally novel, not used in other studies, is the described sandblasting procedure and the use of ultrasonic baths with isopropyl alcohol or distilled water in various combinations.

There has been a long‐lasting controversy regarding possible negative interaction between resin‐based and eugenol‐containing cement. Ribeiro et al. examined the role of eugenol residues on bond strength of total‐ and self‐etching adhesive resin cements to dentine after standardized cleaning procedures (Ribeiro et al., 2011). It was concluded that eugenol residues significantly reduced the bond strength of indirect restorations bonded with both methods. Similar findings were made by Carvalho et al., although the effect was limited to self‐etching resin cement (Carvalho et al., 2007).

This is basically in accordance with present findings, which indicate that when water was used to clean the copings after eugenol containing temporary cement had been used, the TBF of re‐cemented restorations was reduced in comparison to control group C (not exposed to eugenol). True, the present experiment was performed on metal dental implant abutments, not dentine. Nevertheless, this strengthens the hypothesis that under such circumstances, eugenol residues inhibit the polymerization of resin materials and TBS.

On the other hand, the time that the restoration has been exposed to eugenol is another possible reason for different findings. It has been claimed that the TBF is likely to be reduced during the first 24 h after temporary cementation due to a peak release of eugenol. However, after 1–2 weeks this effect disappears (Silva et al., 2011).

Our results also indicate that the use of isopropyl alcohol, either by immersion in an ultrasonic bath or by washing, in combination with sandblasting, is the best way to remove any eugenol residues, thus significantly enhancing the retention (Table 3). This finding is in agreement with de Oliveira et al., who observed the efficacy of different cleaning protocols/agents on the bond strength of dental fiber post and root dentin (de Oliveira et al., 2019). After testing saline solution, acetone, 70% ethanol, and 70% isopropyl alcohol, it was found that with the two alcohols, higher bond strengths were obtained.

Similar findings were made by Safari et al. who investigated the role of implant abutment diameter, cement type, and re‐cementation procedures on TBS of implant‐supported CAD/CAM metal copings (Safari et al., 2018). The conclusion was that resin cement provided the highest TBS and that increasing the abutment diameter also increased TBS. However, re‐cementation with resin cement, after the use of eugenol containing temporary cement, did not cause a significant difference in TBS. Important in this context is that the authors used ultrasonic baths containing ethanol for 15 min, followed by 30 s application of 37% phosphoric acid for removing temporary cement residues.

## 5 CONCLUSION

TBF values for copings luted with resin cement are significantly higher than those luted with temporary cement. If restoration has been previously luted with eugenol containing temporary cement, sandblasting and immersion in an ultrasonic bath or washing with isopropyl alcohol generates the highest TBF values. It seems reasonable to assume that also clinically such procedures ensure the best possible bonding of restorations.

## AUTHOR CONTRIBUTIONS

**Behnz Ebadian**: Supervised the research, designed the research, contributed analysis tools, wrote the paper. **Mohammad Jowkar**: Data collection, performed the analysis, drafted article. **Amin Davoodi**: Wrote the paper, data interpretation. **Amirhossein Fathi**: Data collection. **Mohsen Ziaei**: Data collection. **Einar Berg**: Supervised the writing, Critical revision of the article.

## CONFLICT OF INTEREST

The authors declare no conflict of interest.

## Data Availability

Data are available whenever needed.
